# Clinical performance of decellularized heart valves versus standard tissue conduits: a systematic review and meta-analysis

**DOI:** 10.1186/s13019-020-01292-y

**Published:** 2020-09-18

**Authors:** Steve W. F. R. Waqanivavalagi, Sameer Bhat, Marcus B. Ground, Paget F. Milsom, Jillian Cornish

**Affiliations:** 1 Department of Medicine, Faculty of Medical and Health Sciences, University of Auckland, Grafton, Auckland, 1023 New Zealand; 2 Adult Emergency Department, Auckland City Hospital, Auckland District Health Board, Grafton, Auckland, 1023 New Zealand; 3 Department of Surgery, Faculty of Medical and Health Sciences, University of Auckland, Grafton, Auckland, 1023 New Zealand; 4 Department of Medicine, Dunedin School of Medicine, University of Otago, Dunedin, 9054 New Zealand; 5 Green Lane Cardiothoracic Surgical Unit, Auckland City Hospital, Auckland District Health Board, Grafton, Auckland, 1023 New Zealand

**Keywords:** Decellularization, Tissue engineering, Valve replacement

## Abstract

**Background:**

Valve replacement surgery is the definitive management strategy for patients with severe valvular disease. However, valvular conduits currently in clinical use are associated with significant limitations. Tissue-engineered (decellularized) heart valves are alternative prostheses that have demonstrated promising early results. The purpose of this systematic review and meta-analysis is to perform robust evaluation of the clinical performance of decellularized heart valves implanted in either outflow tract position, in comparison with standard tissue conduits.

**Methods:**

Systematic searches were conducted in the PubMed, Scopus, and Web of Science databases for articles in which outcomes between decellularized heart valves surgically implanted within either outflow tract position of human subjects and standard tissue conduits were compared. Primary endpoints included postoperative mortality and reoperation rates. Meta-analysis was performed using a random-effects model via the Mantel-Haenszel method.

**Results:**

Seventeen articles were identified, of which 16 were included in the meta-analysis. In total, 1418 patients underwent outflow tract reconstructions with decellularized heart valves and 2725 patients received standard tissue conduits. Decellularized heart valves were produced from human pulmonary valves and implanted within the right ventricular outflow tract in all cases. Lower postoperative mortality (4.7% vs. 6.1%; RR 0.94, 95% CI: 0.60–1.47; *P* = 0.77) and reoperation rates (4.8% vs. 7.4%; RR 0.55, 95% CI: 0.36–0.84; *P* = 0.0057) were observed in patients with decellularized heart valves, although only reoperation rates were statistically significant. There was no statistically significant heterogeneity between the analyzed articles (*I*^2^ = 31%, *P* = 0.13 and *I*^2^ = 33%, *P* = 0.10 respectively).

**Conclusions:**

Decellularized heart valves implanted within the right ventricular outflow tract have demonstrated significantly lower reoperation rates when compared to standard tissue conduits. However, in order to allow for more accurate conclusions about the clinical performance of decellularized heart valves to be made, there need to be more high-quality studies with greater consistency in the reporting of clinical outcomes.

## Introduction

Valve replacement surgery is the definitive management strategy for patients with advanced valvular disease [1–3]. However, the conduits used at operation have each faced some limitation precluding their routine clinical use. For example, although mechanical valves are often selected for younger patients because of their long-lasting durability [4, 5], their higher thromboembolic risk means that patients require lifelong anticoagulation, which carries a risk of life-threatening hemorrhage [6–8]. Thus, for women of child-bearing age or the elderly, a bioprosthetic valve might be selected instead, thereby obviating the need for anticoagulants [9]. Despite this advantage, bioprosthetic valves elicit an inflammatory response that leads to progressive calcification, graft failure, and earlier reintervention [8, 10]. This inflammatory reaction is especially profound in children, which is why homografts are often selected as the conduits of choice for pediatric patients [11]. Nevertheless, homografts remain susceptible to graft-versus-host disease, lack regenerative capacity, and are often poorly available [12]. Thus, an ideal valvular prosthesis is yet to be identified [13–16].

The characteristics of an ideal valve replacement prosthesis would be: durability [14, 17, 18], non-immunogenicity [15, 19], non-thrombogenicity [16, 20], capability of growth, self-repair and remodeling [15–19], and lasting the recipient’s lifespan [15]. In an attempt to achieve this goal, tissue-engineered heart valves have been designed using various combinations of the following framework: firstly, the genetic material of the xenograft is removed through a decellularization process that produces an acellular connective tissue scaffold upon which host cells are seeded [21, 22]. The valve is then conditioned in a bioreactor before surgical implantation [23, 24]. It is anticipated that the finished product is a living tissue conduit that meets the requirements of an ideal valve prosthesis.

Unfortunately, early clinical trials of decellularized grafts have produced catastrophic results. A report on the clinical performance of the SynerGraft™ porcine valve implanted within pediatric patients revealed that the graft elicited a strong inflammatory response with fatal consequences soon after implantation [25]. However, these outcomes are not universal. Konertz et al. reported that the Matrix P™ graft, a decellularized porcine xenograft, had a similar physiological performance to the native valve [26]. Furthermore, significant improvements in decellularization technology have been achieved since the complications following graft implantation in humans first attracted concern [27, 28] and decellularized heart valves have since been examined in large animal models and humans with encouraging outcomes [29–31].

Heart valve tissue-engineering has now matured to the point where various products have been brought to clinical trial [27, 32–34]. However, the performance of these conduits is yet to be systematically evaluated. This systematic review and meta-analysis was undertaken to: (1) record, assess and discuss the collective clinical performance of decellularized heart valves compared with standard conduits; (2) establish whether groups that have investigated the clinical utility of decellularized heart valves report similar categories of findings, and, if not; (3) develop a recommendation for outcomes that future groups might consider when publishing the results of their studies.

## Methods

This systematic review was conducted in accordance with the Preferred Reporting Items for Systematic Reviews and Meta-Analyses (PRISMA) guidelines [35]. Two reviewers (S.B. and S.W.) systematically searched medical databases, assessed the titles, abstracts, and full texts of articles, extracted the data, and performed quality assessments using standard quality assessment tools.

### Literature search strategy

PubMed, Scopus and Web of Science electronic databases were searched from their dates of inception to September 2019. The search terms “decellularization”, “acellularization” and “valve” were combined as either keywords or Medical Subject Headings (MeSH) terms using the Boolean operators “AND” and “OR” in the following format: [decellular* OR acellular*] AND valv*. There were no date or geographic restrictions on search results. Titles and abstracts of the retrieved results were independently assessed, and reference lists of the extracted publications were manually reviewed to capture all potentially relevant studies. The full text of each possibly relevant article was independently appraised. Eligible articles were then set aside, and any disagreements were resolved by consensus, prior to finalizing the list of included texts for systematic review.

### Study selection

Articles were selected for inclusion if they were written in English and reported any of the clinical or preclinical outcomes for decellularized heart valves surgically implanted within the outflow tract position of human subjects. Studies were excluded if the decellularization protocol for heart valves was not clearly outlined or if a previously published decellularization protocol was not adequately referenced, there was no control or comparison group, or if the outcomes for the decellularized group of subjects was not separately analyzed. There were no restrictions on study design, study size, date, or geographic location.

Abstracts, book chapters, case reports, conference presentations, retracted articles, editorials, letters to the editor, commentaries, and expert opinions were excluded. Review articles were omitted to avoid publication bias and the duplication of results. Articles were also excluded if the full text could not be sourced.

### Data extraction

Data were recorded in a Microsoft Excel® (Microsoft Corporation, Redmond, Washington, US) proforma spreadsheet. The following agreed data points were recorded from each article: article characteristics (authors, year of publication, study design, and geographic location); basic demographic characteristics (total and decellularized valve sample sizes, gender, age, and mass); indication(s) for, and characteristics of, the decellularized heart valve replacements; the heart valve decellularization protocol employed; and the pre-determined primary and secondary endpoints (Table 1).

**Table 1:** Pre-determined primary and secondary endpoints extracted from eligible full-text articles

Primary endpoints		Secondary endpoints				
Mortality	Reoperations	Transvalvular gradient	Valvular function	Valvular characteristics	Histological commentary	Adverse events
30-day	30-day	Overall	Insufficiency	Diameter	Fibroproliferation	Infective endocarditis OR freedom from infective endocarditis
1-year	1-year		Stenosis	Area	Recellularization	Valve thrombosis OR freedom from valve thrombosis
Overall rate OR freedom from mortality	Overall rate OR freedom from reoperations		Explantation rate OR freedom from explantation	Calcification	Antibody/humoral immune response	
	Time to reoperation		Rate of dysfunction OR freedom from dysfunction	Cusp mobility		
			Failure rate OR freedom from failure	Cusp retraction		

### Quality appraisal

Appraisals of the methodological quality of included observational cohort studies were performed using the Newcastle-Ottawa Quality Assessment Scale [36]. This scale assigns a score to each of three quality assessment domains using a ‘star system’. Awarding a star indicates the presence of a particular study design characteristic (refer to Additional file 1). High-quality studies were those achieving seven or more stars, medium-quality were those achieving four to six stars, and low-quality were those achieving fewer than four stars.

The risk of bias within the included non-randomized interventional studies was identified using the Risk of Bias In Non-Randomized Studies of Interventions (ROBINS-I) tool [37]. The ROBINS-I tool divides each study into seven bias domains. Each of these bias domains was classified as at either low, moderate, serious, or critical risk of bias. An overall judgment of the risk of bias for each study was then made using an identical assessment rubric. Studies with an overall “low” risk of bias were considered equivalent to a well-performed randomized controlled trial (RCT).

### Statistical analysis

Meta-analysis was performed for the primary endpoints (postoperative mortality and reoperation rates) using the meta package with R software (Version 4.0.0; R Foundation for Statistical Computing, Vienna, Austria). The Mantel-Haenszel method was used to calculate the pooled relative risk (RR) and 95% confidence interval (CI) for the binary primary endpoints (mortality versus no mortality and reoperation versus no reoperation). Statistical heterogeneity between studies was evaluated using the Cochran Q test and then quantified using the *I*^2^ test statistic [38]. We opted for a random-effects rather than a fixed-effects model, due to the expected level of heterogeneity between the studies included in the meta-analysis. Publication biases were assessed using Egger’s regression test and by generating funnel plots. Sensitivity analyses were performed by excluding studies at moderate or serious risk of bias, which may have had large heterogeneity, and studies with very large sample sizes, which may have disproportionately influenced the pooled summary estimate. Secondary endpoints and basic demographic data were synthesized qualitatively, using probability (P) values, total numbers and percentages where appropriate. *P*-values < 0.05 were considered statistically significant for all reported outcomes.

## Results

### Quantity of evidence

**Fig. 1:**
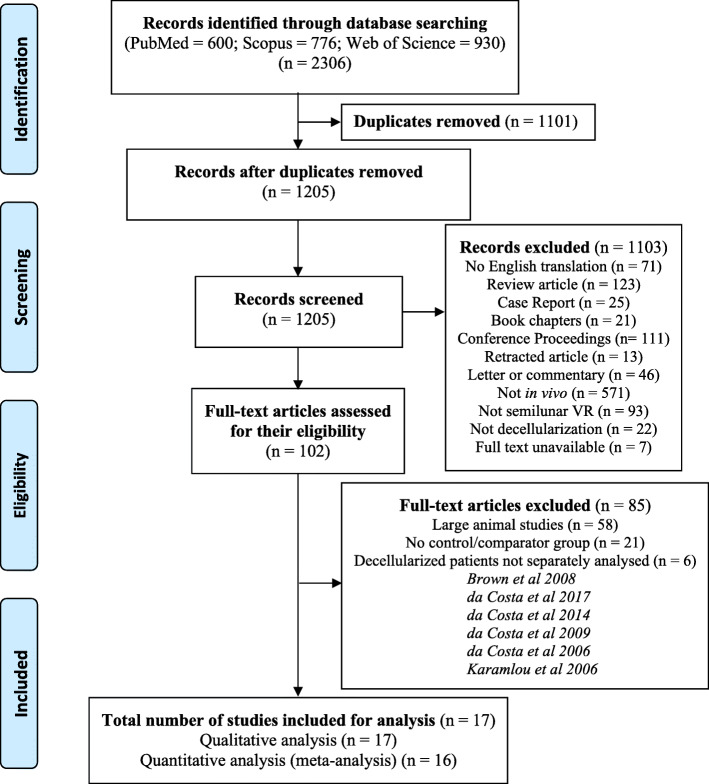
PRISMA flow diagram demonstrating the full-text article selection process

The search of medical databases revealed 2306 abstracts, from which 102 full-text articles were assessed for their eligibility and inclusion (PRISMA Diagram: Fig. 1). In total, 17 full-text articles were included for qualitative analysis. All but one study were included for meta-analysis of primary endpoints [39]. The characteristics of each of these articles are provided in Table 2.

**Table 2:** Characteristics of eligible full-text articles

Authors	Year	Study design	Retrospective/prospective	Geographic location
Bechtel et al. [39]	2005	Interventional, non-randomized controlled trial	Prospective	Germany
Bechtel et al. [40]	2008	Interventional, non-randomized controlled trial	Prospective	Germany
Bibevski et al. [41]	2017	Observational cohort study	Retrospective	United States of America
Boethig et al. [42]	2019	Observational cohort study	Prospective	Germany
Brown et al. [43]	2010	Observational cohort study	Retrospective	United States of America
Brown et al. [44]	2011	Observational cohort study	Retrospective	United States of America
Burch et al. [45]	2010	Observational cohort study	Retrospective	United States of America
Cebotari et al. [46]	2011	Interventional, non-randomized controlled trial	Prospective	Germany
da Costa et al. [47]	2005	Interventional, non-randomized controlled trial	Prospective	Brazil
da Costa et al. [33]	2007	Interventional, non-randomized controlled trial	Prospective	Brazil
da Costa et al. [48]	2018	Interventional, non-randomized controlled trial	Prospective	Brazil
Etnel et al. [49]	2018	Interventional, non-randomized controlled trial	Prospective	Netherlands
Konuma et al. [50]	2009	Observational cohort study	Retrospective	United States of America
Ruzmetov et al. [51]	2012	Observational cohort study	Retrospective	United States of America
Sarikouch et al. [34]	2016	Interventional, non-randomized controlled trial	Prospective	Germany
Sievers et al. [52]	2003	Interventional, non-randomized controlled trial	Prospective	Germany
Tavakkol et al. [53]	2005	Observational cohort study	Retrospective	United States of America

The articles selected for data analysis were conducted across four countries, most frequently within the United States of America, and they spanned a 16-year interval, from 2003 to 2019. The published experiences of outflow tract reconstructions using decellularized heart valves were equally divided between interventional and observational cohort studies (*n* = 9 and *n* = 8 studies, respectively). The observational cohort studies were largely retrospective (*n* = 7 studies), with a single study conducted prospectively (Table 2). There were no RCT.

### Quality of evidence

All included observational cohort studies were judged as high-quality, based on their Newcastle-Ottawa Quality Assessment scores (refer to Additional file 2).

Only two of the nine (~ 22%) included non-randomized interventional studies were judged as being at “low” risk of bias, and hence, were comparable to a well-performed RCT [48, 49]. The remaining studies were judged as at either “moderate” or “serious” risk of bias (*n* = 4 and *n* = 3 studies, respectively), with such judgment being made principally on the basis of either bias due to confounding or missing data [33, 39, 40] (refer to Additional file 3).

### Basic demographics

#### Population descriptors

Outflow tract reconstructions were performed in 4143 patients, of whom 1418 (34.2%) patients received decellularized heart valves and 2725 (65.8%) patients received standard tissue conduits (refer to population specific data provided in Table 3).

**Table 3:** Population descriptors of eligible full-text articles

Authors	Total sample size	Standard tissue conduit sample size	Decellularized valve sample size	Sex (M;F)^a^	[Median age]; mean age ± SD (range), years^a^	[Median mass]; mean mass ± SD (range), kg^a^
Bechtel et al. 2005 [39]	69	47	22	18;4	37.4 ± 10.2	
Bechtel et al. 2008 [40]	72	49	23	19;4	37.0 ± 10.8	
Bibevski et al. 2017 [41]	287	124	163	107;56	17.3 ± 16.4 (0.005–74)	45.8 ± 36.3 (2–126)
Boethig et al. 2019 [42]	705	470	235	136;99	19.1 ± 12.8	
Brown et al. 2010 [43]	1588	1246	342	229;113	23.9 ± 12.6 (0–69.6)	
Brown et al. 2011 [44]	63	34	29		[22]; 28.6 ± 16.0 (0.25–58)	[81]; 79.3 ± 28.4 (5–126)
Burch et al. 2010 [45]	94	47	47		[9.11]; 9.91 ± 8.08 (0.011–30.03)	[25]; 33 ± 26 (3–90)
Cebotari et al. 2011 [46]	114	76	38	16;22	16.4 ± 11.4	
da Costa et al. 2005 [47]	20	9	11	5;6	23.0 ± 9.04 (9–37)	
da Costa et al. 2007 [33]	136	68	68	48;20	30.3 ± 11.2 (9–56)	
da Costa et al. 2018 [48]	188	94	94	74;20	[34.0]	
Etnel et al. 2018 [49]	260	130	130	93;37	[28]	
Konuma et al. 2009 [50]	82	41	41		5.5 ± 7.4	20.5 ± 22.0
Ruzmetov et al. 2012 [51]	100	61	39	25;14	19.2 ± 17.2 (0.02–72)	54.7 ± 40.4 (2.8–167)
Sarikouch et al. 2016 [34]	279	186	93	58;35	15.8 ± 10.2	
Sievers et al. 2003 [52]	34	17	17	13;4	39.7 ± 9.7	
Tavakkol et al. 2005 [53]	52	26	26		5.1 ± 5.4	20.5 ± 22.7

Blank cells correspond with data points that were not reported in the respective article

*F* female, kg kilogram, M male, *SD* standard deviation

^a^ These data relate to the decellularized heart valve patient population

#### Semilunar heart valve replacement descriptors

The indications for outflow tract reconstruction surgery were reported in 11 of the 17 included articles. In descending order of frequency, these indications were: congenital heart disease, mixed (stenotic and regurgitant) valvular disease, valvular regurgitation, valvular stenosis, and infective endocarditis (Additional file 4).

The decellularized heart valves comprised pulmonary valve tissue obtained from human donors and implantation of the grafts was restricted to human recipients in all studies. Decellularized pulmonary valves were implanted within the right ventricular outflow tract in each article. Thus, no cases of left ventricular outflow tract reconstruction were reported (Table 4).

**Table 4:** Decellularized heart valve characteristics in eligible full-text articles

Authors	Donor species	Donor tissue/valve type	Recipient species	Recipient site	Number of patients
Bechtel et al. 2005^a^ [39]	Human	PV	Human	RVOT	22
Bechtel et al. 2008^a^ [40]	Human	PV	Human	RVOT	23
Bibevski et al. 2017^a^ [41]	Human	PV	Human	RVOT	163
Boethig et al. 2019 [42]	Human	PV	Human	RVOT	235
Brown et al. 2010^a^ [43]	Human	PV	Human	RVOT	342
Brown et al. 2011^a^ [44]	Human	PV	Human	RVOT	29
Burch et al. 2010^a^ [45]	Human	PV	Human	RVOT	47
Cebotari et al. 2011 [46]	Human	PV	Human	RVOT	38
da Costa et al. 2005 [47]	Human	PV	Human	RVOT	11
da Costa et al. 2007^b^ [33]	Human	PV	Human	RVOT	68
da Costa et al. 2018^c^ [48]	Human	PV	Human	RVOT	94
Etnel et al. 2018^c^ [49]	Human	PV	Human	RVOT	130
Konuma et al. 2009^a^ [50]	Human	PV	Human	RVOT	41
Ruzmetov et al. 2012 [51]	Human	PV	Human	RVOT	39
Sarikouch et al. 2016 [34]	Human	PV	Human	RVOT	93
Sievers et al. 2003 [52]	Human	PV	Human	RVOT	17
Tavakkol et al. 2005^a^ [53]	Human	PV	Human	RVOT	26

*PV* pulmonary valve, *RVOT* right ventricular outflow tract

^a^CryoValve SynerGraft (CryoLife Inc., Kennesaw, Georgia, US) pulmonary homograft

^b^Pulmonary homografts were provided by the Human Heart Valve Bank (Hospital Santa Casa de Misericórdia, Curitiba, BR)

^c^Pulmonary homografts were obtained at the Multi-Tissue Bank (Pontifical Catholic University of Paraná, Curitiba Campus, Curitiba, BR)

#### Decellularization protocols for semilunar heart valves

The decellularization protocol for pulmonary valve homografts was adequately described or referenced in all articles. Three articles described novel decellularization processes that typically comprised: 0.9% sodium chloride, the detergents sodium deoxycholate and sodium dodecylsulfate, penicillin and streptomycin as the sole combination of antimicrobial agents, and incubation at temperatures ranging from − 150 °C to 20 °C [33, 34, 46]. In the remainder of articles, valves were decellularized using one of three previously patented or published protocols [27, 28, 54] (Table 5). The protocol developed by CryoLife Inc. (CryoLife Inc., Kennesaw, Georgia, US) was employed in 10 of the remaining 14 articles [54].

**Table 5:** Decellularization protocol for replacement heart valves in eligible full-text articles

Authors	Decellularization components and conditions			
	Detergent(s)	Enzyme(s)	Antimicrobial agent(s)	Temperature
Bechtel et al. 2005^a^ [39]	Hypotonic sterile water solution (treatment); multi-day washout period with isotonic neutral buffer solution (unspecified)	Ribonuclease + deoxyribonuclease	Antibiotic + antimycotic (unspecified)	
Bechtel et al. 2008^a^ [40]	Hypotonic sterile water solution (treatment); multi-day washout period with isotonic neutral buffer solution (unspecified)	Ribonuclease + deoxyribonuclease	Antibiotic + antimycotic (unspecified)	
Bibevski et al. 2017^a^ [41]	Hypotonic sterile water solution (treatment); multi-day washout period with isotonic neutral buffer solution (unspecified)	Ribonuclease + deoxyribonuclease	Antibiotic + antimycotic (unspecified)	
Boethig et al., 2019^b^ [42]	0.5% sodium deoxycholate (Sigma Chemical Company, St Louis, Missouri, USA) + 0.5% sodium dodecylsulfate (Carl Roth GmbH, Karlsruhe, Germany); 0.9% NaCl (washing)		Penicillin-streptomycin (100 IU/mL)^e^	20 °C (treatment); 4 °C (storage)
Brown et al. 2010^a^ [43]	Hypotonic sterile water solution (treatment); multi-day washout period with isotonic neutral buffer solution (unspecified)	Ribonuclease + deoxyribonuclease	Antibiotic + antimycotic (unspecified)	
Brown et al. 2011^a^ [44]	Hypotonic sterile water solution (treatment); multi-day washout period with isotonic neutral buffer solution (unspecified)	Ribonuclease + deoxyribonuclease	Antibiotic + antimycotic (unspecified)	
Burch et al. 2010^a^ [45]	Hypotonic sterile water solution (treatment); multi-day washout period with isotonic neutral buffer solution (unspecified)	Ribonuclease + deoxyribonuclease	Antibiotic + antimycotic (unspecified)	
Cebotari et al. 2011 [46]	0.5% sodium deoxycholate + 0.5% sodium dodecylsulfate (treatment); 0.9% NaCl (washing)		Penicillin-streptomycin (100 IU/mL)^e^	20 °C (treatment); 4 °C (storage)
da Costa et al. 2005^c^ [47]	1 to 2% sodium deoxycholic acid (treatment); PBS in decreasing concentrations (rinsing); 70% ethanol; pulsating PBS (repeating rinsing)			37 °C (storage for 24 h); 20 °C (treatment)
da Costa et al. 2007 [33]	10% dimethyl sulphoxide + 10% fetal bovine serum; 0.9% NaCl (thawing) + 10% fetal bovine serum (gradual dilution); continuous shaking over 24 h with either 1% sodium deoxycholic acid + 80% ethanol OR 0.1% sodium dodecylsulfate; nutrient solution (harvesting)		Cefoxitin (240 μg/mL) + lincomycin (120 μg/mL) + vancomycin (50 μg/mL) + polymyxin B (100 μg/mL)	4 °C (treatment); Storage within −150 °C liquid nitrogen vapour; 42 to 50 °C (thawing)
da Costa et al. 2018^d^ [48]	0.1% sodium dodecylsulfate (treatment); PBS (storage)			37 °C (shaking); 4 °C (storage up to 90 days)
Etnel et al. 2018^d^ [49]	0.1% sodium dodecylsulfate (treatment); PBS (storage)			37 °C (shaking); 4 °C (storage up to 90 days)
Konuma et al. 2009^a^ [50]	Hypotonic sterile water solution (treatment); multi-day washout period with isotonic neutral buffer solution (unspecified)	Ribonuclease + deoxyribonuclease	Antibiotic + antimycotic (unspecified)	
Ruzmetov et al. 2012^a^ [51]	Hypotonic sterile water solution (treatment); multi-day washout period with isotonic neutral buffer solution (unspecified)	Ribonuclease + deoxyribonuclease	Antibiotic + antimycotic (unspecified)	
Sarikouch et al. 2016 [34]	0.5% sodium deoxycholate (Sigma Chemical Company, St Louis, Missouri, USA) + 0.5% sodium dodecylsulfate (Carl Roth GmbH, Karlsruhe, Germany) (treatment); PBS (6 cycles of washing, 12 h each)		Penicillin-streptomycin (100 IU/mL)^e^	4 °C (storage)
Sievers et al. 2003^a^ [52]	Hypotonic sterile water solution (treatment); multi-day washout period with isotonic neutral buffer solution (unspecified)	Ribonuclease + deoxyribonuclease	Antibiotic + antimycotic (unspecified)	
Tavakkol et al. 2005^a^ [53]	Hypotonic sterile water solution (treatment); multi-day washout period with isotonic neutral buffer solution (unspecified)	Ribonuclease + deoxyribonuclease	Antibiotic + antimycotic (unspecified)	

*°C* degrees celsius, *Ltd.* limited liability company, *mL* milliliter, *NaCl* sodium chloride, *PBS* phosphate-buffered saline

^a^Pulmonary homograft decellularized according to the SynerGraft™ treatment protocol (CryoLife Inc., Kennesaw, Georgia, US) [54]

^b^Decellularization of pulmonary homograft performed at corlife oHG (corlife oHG, Hannover Medical School, Hannover, DE)

^c^Pulmonary homograft decellularized according to the patented process developed by AutoTissue Ltd. (AutoTissue GmbH, Berlin, DE) [27]

^d^Pulmonary homograft decellularized according to the patented process developed by Tissue Regenix Ltd. (Tissue Regenix Ltd., Leeds, Yorkshire, UK) [28]

^e^Penicillin-streptomycin solution manufactured by Cedarlane Laboratories (PenStrep, Cedarlane Laboratories, Burlington, Ontario, CA)

### Primary endpoints (Additional file 5)

#### Postoperative mortality

**Fig. 2:**
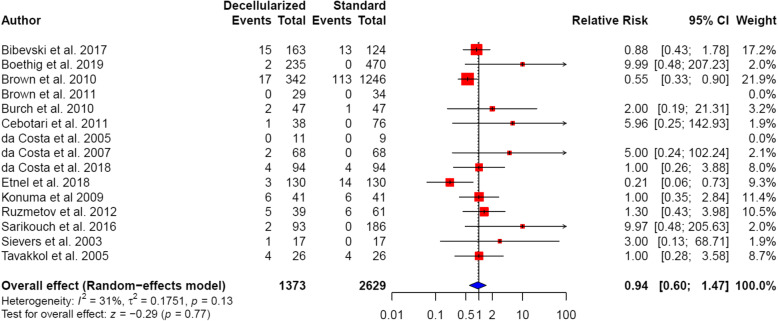
Forest plot of postoperative mortality rates following outflow tract reconstruction surgery with decellularized heart valves versus standard tissue conduits. Pooled summary estimates are shown as relative risks (RR) with their 95% confidence intervals (CI)

Fifteen of the included studies reported postoperative mortality rates. Two of the studies demonstrated statistically significant results, with lower postoperative mortality rates in patients with decellularized heart valves [43, 49]. Two included studies did not report any instances of mortality and thus did not contribute to the pooled summary estimate [44, 47]. Overall, lower postoperative mortality rates were observed in patients with decellularized heart valves, when compared to patients with standard tissue conduits, although these findings were not statistically significant (4.7% vs. 6.1%; pooled RR 0.94, 95% CI: 0.60 to 1.47; *P* = 0.77; Fig. 2). There was no significant heterogeneity between the analyzed studies (*I*^2^ = 31%; *P* = 0.13).

#### Reoperation

**Fig. 3:**
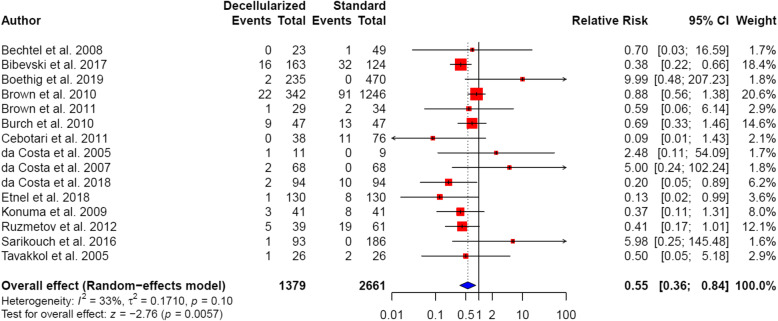
Forest plot of reoperation rates following outflow tract reconstruction surgery with decellularized heart valves versus standard tissue conduits. Pooled summary estimates are shown as relative risks (RR) with their 95% confidence intervals (CI)

Fifteen included studies reported reoperation rates. Three of the studies demonstrated statistically significant results, with lower reoperation rates observed for patients with decellularized heart valves [41, 48, 49]. Overall, significantly lower reoperation rates were observed in patients with decellularized heart valves, when compared to patients with standard tissue conduits (4.8% vs. 7.4%; pooled RR 0.55, 95% CI: 0.36 to 0.84; *P* = 0.0057; Fig. 3). There was no significant heterogeneity between the analyzed studies (*I*^2^ = 33%; *P* = 0.10).

#### Publication bias

**Fig. 4:**
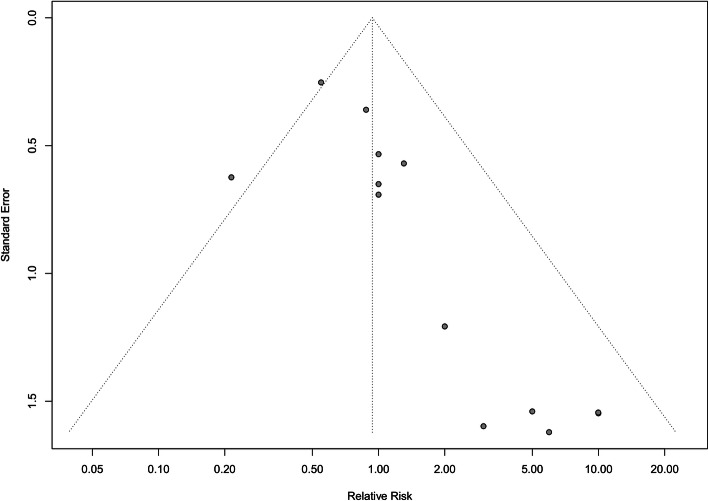
Funnel plot for the assessment of publication bias of postoperative mortality with decellularized heart valves versus standard tissue conduits

**Fig. 5:**
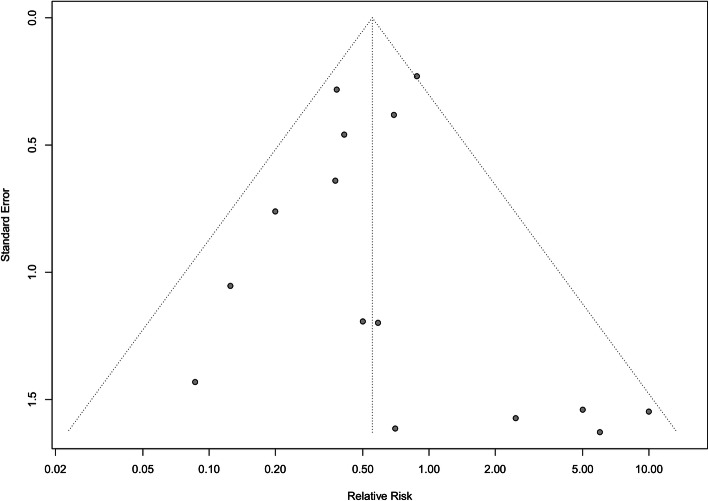
Funnel plot for the assessment of publication bias of reoperation rates with decellularized heart valves versus standard tissue conduits

Funnel plot asymmetry was used to assess publication bias for postoperative mortality rates and reoperation rates within the analyzed studies (Figs. 4 and 5 respectively). Egger’s regression test demonstrated significant publication bias for postoperative mortality rates (*p* = 0.004), with no publication bias observed for reoperation rates (*p* = 0.86).

#### Sensitivity analysis

For the purposes of this sensitivity analysis, six studies were excluded from the original analysis because they were either at moderate or serious risk of bias (*n* = 5 studies) [33, 34, 40, 46, 47, 52] or had a disproportionately large sample size (*n* = 1 study) [43]. Nine studies were included in the final sensitivity analysis of postoperative mortality rates and reoperation rates [41, 42, 44, 45, 48–51, 53]. In comparison to standard tissue conduits, decellularized heart valves were associated with lower postoperative mortality (pooled RR 0.90, 95% CI: 0.55 to 1.46; *P* = 0.66; Additional file 6) and significantly lower reoperation rates (pooled RR 0.44, 95% CI: 0.30 to 0.63; *P* < 0.0001; Additional file 7). There was no significant heterogeneity between the analyzed studies (*I*^2^ = 20%, *P* = 0.27 and *I*^2^ = 5%, *P* = 0.39 respectively).

### Secondary endpoints

Transvalvular gradients were reported in all 17 articles. For patients with decellularized heart valves, 13 articles described either a reduction in the peak transvalvular gradient or a higher freedom from raised transvalvular gradients. Six of these articles, including the largest study by Brown et al., reached statistical significance (*p* < 0.05) [41, 43, 46, 47, 49, 53]. In the four remaining articles, there was either an increase in the peak transvalvular gradient or lower freedom from raised transvalvular gradients. Only Bechtel and colleagues demonstrated the finding to be statistically significant (*p* = 0.049) [40].

The remainder of the secondary endpoints were irregularly reported. These endpoints included: valvular insufficiency [41, 53], valvular stenosis [43, 48, 49], conduit explantation [34], dysfunction [41, 48, 49, 51], and failure rates [43, 51], valvular diameter [39], area [39, 52], cusp mobility and retraction [33, 46], histological valvular analysis [33, 39, 46, 47], and adverse events such as infective endocarditis [34, 42, 43, 48] and valve thrombosis [43].

## Discussion

This systematic review and meta-analysis included 17 studies, which consisted mostly of small studies, with few at low risk of bias. We found that patients with decellularized heart valves implanted within the right ventricular outflow tract had significantly lower reoperation rates and transvalvular gradients, when compared to patients with standard tissue conduits. In addition, the meta-analysis also demonstrated lower postoperative mortality rates among patients with decellularized heart valves, although this result was not statistically significant.

Tissue-engineered heart valves have the potential to revolutionize valve replacement surgery should they be able to overcome the limitations faced by prostheses in current clinical use [4–12]. Xenografts, unlike homografts, would serve as the ideal donor tissue type because of their virtually limitless availability [12]. It is therefore unfortunate that no studies of xenografts were included in this review. Nonetheless, during the screening of full-text articles, 40 studies conducted using decellularized xenograft heart valves were identified but were excluded from the subsequent analysis because they were either not conducted in human subjects (*n* = 29 studies) or did not have a comparator group (*n* = 11 studies) and thus did not meet our inclusion criteria of human trials with a comparison group.

The aortic valve tends to require surgical intervention more frequently than the pulmonary valve because of higher rates of valvular dysfunction [55]. Therefore, the optimal tissue-engineered heart valve must be capable of withstanding the greater pressure demands of the left heart. None of the 17 studies included in this review described the experiences of decellularized valves in the left ventricular outflow tract position. However, during the screening of full-text articles, 15 studies of left ventricular outflow tract reconstructions were identified, although these texts were excluded from the subsequent analysis because their performance was either evaluated in non-human subjects (*n* = 10 studies) or they lacked a comparator group (*n* = 5 studies).

Despite the potential benefits of achieving a decellularized conduit for aortic valve replacement, many international groups have instead elected to focus on designing a graft for the pulmonary valve position because the native pulmonary valve may be transferred to the aortic position during the Ross procedure [27]. Although the Ross procedure has been life-changing for many, especially younger, patients, it remains a more complex operation than an aortic valve replacement [56]. Thus, the design of a prosthetic graft that is specifically intended for the left heart remains a priority.

In addition, a recent concept being explored in heart valve tissue-engineering is construct recellularization using autologous cells [32]. In essence, a decellularized construct is repopulated with the recipient’s cells and then conditioned within a bioreactor ahead of surgical implantation [23, 24]. None of the studies assessed in this article combined a recellularization component with decellularization in their tissue-engineering technology. Much work is being undertaken to explore the capabilities of tissue-engineered scaffolds that are produced from decellularized xenografts and then recellularized using the future host’s autologous cells [23, 24, 32]. Such a graft is expected to be living and thus have the potential to grow, repair, and remodel in situ [15–19].

There are several limitations with this review. Firstly, seven of the included studies were of low quality, with missing data or a lack of confounding being the predominant causes. Secondly, it is significant that only one study of xenograft performance within the left ventricular outflow tract position was identified during the systematic search [57]. Perhaps it is the catastrophic experiences of early xenograft studies that have resulted in the few, low-quality investigations of xenografts in human subjects [25, 58–61]. However, with further development in tissue-engineering technology, the in vitro performance of xenografts could excel to the point where they are brought to clinical trial in large, high-quality studies. It is hypothesized that reducing the immunogenicity of the donor construct is central to achieving this goal. Osmotic shock and the use of enzymes that denature genetic material are useful for this process [15, 16, 20]. Lastly, it is noteworthy that only seven studies incorporated detergents in their decellularization protocol [33, 34, 42, 46–49], because detergent-use has also proven highly effective when incorporated in decellularization protocols [13, 14, 17].

The number of permutations that affects the successfulness of a decellularization process is extensive. For example, small changes in variables such as the detergent combination or concentration, and the duration and temperature of incubation can have a profound impact on the effectiveness of a given protocol. Therefore, tissue-engineering technology should not be dismissed while ongoing laboratory work is being undertaken to refine and further develop decellularization protocols.

## Conclusions

The published experiences of decellularized heart valves implanted within the right ventricular outflow tract of human subjects are typically small studies, in which the available data demonstrate significantly lower reoperation rates and transvalvular gradients, when compared to standard tissue conduits. No studies of tissue-engineered xenografts or conduits implanted within the left ventricular outflow tract were able to be included in this review. Only seven studies incorporated detergents in their decellularization protocol and no studies examined a recellularization component. Thus, the exhaustive clinical potential of tissue-engineered heart valves remains to be elucidated. Future studies should rather aim to investigate the clinical utility of left ventricular outflow tract reconstructions using decellularized heart valves implanted within human subjects. In future, it is also recommended that studies within the heart valve tissue-engineering field consistently report standardized outcomes such as 30-day and one-year postoperative: mortality rates, reoperation rates, transvalvular gradients, valvular dysfunction, diameter and area, in addition to being of higher quality in terms of their design. The adoption of a standardized set of outcomes would allow for more accurate conclusions, particularly regarding patient safety, to be drawn about the undoubtable progress in development of decellularized heart valves and their resultant clinical performance.

## Supplementary information

**Additional file 1.** Criteria for awarding stars using the Newcastle-Ottawa Quality Assessment Scale [36].

**Additional file 2.** Methodological quality assessment for included full-text observational cohort studies [36].

**Additional file 3.** Risk of bias assessment for included full-text non-randomized interventional studies [37].

**Additional file 4.** Indications for outflow tract reconstructions in eligible full-text articles.

**Additional file 5.** Primary endpoints (postoperative mortality and reoperations) in eligible full-text articles.

**Additional file 6.** Forest plot of postoperative mortality rates following outflow tract reconstruction surgery with decellularized heart valves versus standard tissue conduits. Studies at moderate or serious risk of bias and with a disproportionately large sample size have been excluded for the purposes of this sensitivity analysis. Pooled summary estimates are shown as relative risks (RR) with their 95% confidence intervals (CI).

**Additional file 7.** Forest plot of reoperation rates following outflow tract reconstruction surgery with decellularized heart valves versus standard tissue conduits. Studies at moderate or serious risk of bias and with a disproportionately large sample size have been excluded for the purposes of this sensitivity analysis. Pooled summary estimates are shown as relative risks (RR) with their 95% confidence intervals (CI).

## Data Availability

The datasets used and analyzed during the current study are available from the corresponding author on reasonable request.

## Abbreviations

CI: Confidence Interval
MeSH: Medical Subject Headings
*P*-value: Probability value
PRISMA: Preferred Reporting Items for Systematic Reviews and Meta-Analyses
RCT: Randomized Controlled Trial
ROBINS-I: Risk of Bias In Non-Randomized Studies of Interventions
RR: Relative Risk
