# Supplementary material for: Clinical performance of decellularized heart valves versus standard tissue conduits: a systematic review and meta-analysis
Source: J Cardiothorac Surg. 2020 Sep 18;15:260. doi: 10.1186/s13019-020-01292-y (PMC7501674; doi:10.1186/s13019-020-01292-y)
Supplement: Supplementary file 1 — Additional file 1. Criteria for awarding stars using the Newcastle-Ottawa Quality Assessment Scale [36]. [file 13019_2020_1292_MOESM1_ESM.docx]

| **Additional File 1.** Criteria for awarding stars using the Newcastle-Ottawa Quality Assessment Scale ^36^ | | | |
| --- | --- | --- | --- |
| **Quality Assessment Domain** | | **Criteria acceptable (★ awarded)** | **Criteria unacceptable (★ not awarded)** |
| Selection of study cohorts | Representativeness of the exposed cohort | Truly or partially representative (e.g. population-based) | Not representative (selected group of users e.g. volunteers) OR description of cohort derivation not provided |
|  | Selection of the non-exposed cohort | Drawn from an identical setting as the exposed cohort | Drawn from a different setting to the exposed cohort OR description of cohort derivation not provided |
|  | Ascertainment of exposure | Secure records (e.g. medical/surgical records) OR structured interview | Self-reported information OR no description provided |
|  | Demonstration that outcome of interest was not present at study commencement | Yes | No |
| Comparability of study cohorts | Comparability of cohorts on basis of study design or analysis | One (or more) factors controlled by the study design or analysis | No factors controlled by the study design or analysis |
| Ascertainment of outcome of interest | Assessment of outcome | Independent blind assessment OR linkage to secure records | Self-reported information OR no description provided |
|  | Sufficient follow-up duration to capture outcomes | Yes (adequate follow-up period based on the particular outcome of interest) | No |
|  | Adequacy of follow-up | Complete follow-up for all subjects (no missing data) OR small numbers lost to follow-up and description provided for those lost to follow-up | Large numbers lost to follow-up OR no description provided OR no statement regarding missing data |
