# Supplementary material for: Clinical performance of decellularized heart valves versus standard tissue conduits: a systematic review and meta-analysis
Source: J Cardiothorac Surg. 2020 Sep 18;15:260. doi: 10.1186/s13019-020-01292-y (PMC7501674; doi:10.1186/s13019-020-01292-y)
Supplement: Supplementary file 2 — Additional file 2. Methodological quality assessment for included full-text observational cohort studies [36]. [file 13019_2020_1292_MOESM2_ESM.docx]

| **Additional File 2.** Methodological quality assessment for included full-text observational cohort studies ^36^ | | | | | | | | | |
| --- | --- | --- | --- | --- | --- | --- | --- | --- | --- |
| **Authors** | **Quality Assessment Domain** | | | | | | | | **Total (/9★)** |
|  | **Selection of study groups** | | | | **Comparability of study groups**^†^ | **Ascertainment of outcome of interest** | | |  |
|  | Representativeness of exposed cohort (★) | Selection of non-exposed cohort (★) | Ascertainment of exposure (★) | Demonstration that outcome was not present at study commencement (★) | Comparability of cohorts on basis of study design or analysis (★★) | Assessment of outcome (★) | Follow-up duration (★) | Adequacy of follow-up (★) |  |
| Bibevski et al. 2017 ^48^ | ★ | ★ | ★ | ★ |  | ★ | ★ | ★ | 7★ |
| Boethig et al. 2019 ^50^ | ★ | ★ | ★ | ★ | ★★ |  |  | ★ | 7★ |
| Brown et al. 2010 ^45^ | ★ | ★ | ★ | ★ |  | ★ | ★ | ★ | 7★ |
| Brown et al. 2011 ^47^ | ★ | ★ | ★ | ★ |  | ★ | ★ | ★ | 7★ |
| Burch et al. 2010 ^51^ | ★ | ★ | ★ | ★ | ★★ | ★ | ★ |  | 8★ |
| Konuma et al. 2009 ^52^ | ★ | ★ | ★ | ★ | ★★ | ★ | ★ |  | 8★ |
| Ruzmetov et al. 2012 ^53^ | ★ | ★ | ★ | ★ |  | ★ | ★ | ★ | 7★ |
| Tavakkol et al. 2005 ^54^ | ★ | ★ | ★ | ★ | ★★ | ★ |  |  | 7★ |
| ^†^Two stars may be awarded depending on the number of factors being controlled based on the study design or analysis, to enable comparability between the exposed and non-exposed cohorts. One star is awarded if only a single factor is controlled, and two stars are awarded if two or more factors are controlled. | | | | | | | | | |
