# Supplementary material for: Clinical performance of decellularized heart valves versus standard tissue conduits: a systematic review and meta-analysis
Source: J Cardiothorac Surg. 2020 Sep 18;15:260. doi: 10.1186/s13019-020-01292-y (PMC7501674; doi:10.1186/s13019-020-01292-y)
Supplement: Supplementary file 3 — Additional file 3. Risk of bias assessment for included full-text non-randomized interventional studies [37]. [file 13019_2020_1292_MOESM3_ESM.docx]

| **Additional File 3.** Risk of bias assessment for included full-text non-randomized interventional studies ^37^ | | | | | | | | |
| --- | --- | --- | --- | --- | --- | --- | --- | --- |
| **Authors** | **Bias due to confounding** | **Bias in selection of participants** | **Bias in classification of interventions** | **Bias due to deviations from intended interventions** | **Bias due to missing data** | **Bias in measurement of outcomes** | **Bias in selection of the reported result** | **Overall risk of bias judgement** |
| Bechtel et al. 2005 ^39^ | Serious risk | Low risk | Low risk | Low risk | Low risk | Moderate risk | Moderate risk | Serious risk |
| Bechtel et al. 2008 ^42^ | Serious risk | Low risk | Low risk | Low risk | Low risk | Moderate risk | Low risk | Serious risk |
| Cebotari et al. 2011 ^43^ | Low risk | Low risk | Low risk | Low risk | Moderate risk | Low risk | Low risk | Moderate risk |
| da Costa et al. 2005 ^46^ | Low risk | Low risk | Low risk | Low risk | Low risk | Moderate risk | Moderate risk | Moderate risk |
| da Costa et al. 2007 ^33^ | Moderate risk | Low risk | Moderate risk | Low risk | Serious risk | Moderate risk | Moderate risk | Serious risk |
| da Costa et al. 2018 ^40^ | Low risk | Low risk | Low risk | Low risk | Low risk | Low risk | Low risk | Low risk |
| Etnel et al. 2018 ^41^ | Low risk | Low risk | Low risk | Low risk | Low risk | Low risk | Low risk | Low risk |
| Sarikouch et al. 2016 ^34^ | Low risk | Low risk | Moderate risk | Low risk | Low risk | Moderate risk | Low risk | Moderate risk |
| Sievers et al. 2003 ^49^ | Moderate risk | Low risk | Low risk | Low risk | Low risk | Moderate risk | Low risk | Moderate risk |
