# Supplementary material for: Clinical performance of decellularized heart valves versus standard tissue conduits: a systematic review and meta-analysis
Source: J Cardiothorac Surg. 2020 Sep 18;15:260. doi: 10.1186/s13019-020-01292-y (PMC7501674; doi:10.1186/s13019-020-01292-y)
Supplement: Supplementary file 4 — Additional file 4. Indications for outflow tract reconstructions in eligible full-text articles. [file 13019_2020_1292_MOESM4_ESM.docx]

| **Additional File 4.** Indications for outflow tract reconstructions in eligible full-text articles | | |
| --- | --- | --- |
| **Indication for outflow tract reconstruction** | **Number of patients** | **Reference text(s)** |
| Congenital heart disease ^†^ | 542 | Bibevski et al.^48^; Boethig et al.^50^; Brown et al.^45^; Cebotari et al.^43^; Ruzmetov et al.^53^; Sarikouch et al.^34^ |
| Mixed (stenotic and regurgitant) valvular disease | 231 | Bibevski et al.^48^; Boethig et al.^50^; Cebotari et al.^43^; da Costa et al.^33,40^; Etnel et al.^41^; Sarikouch et al.^34^; Sievers et al.^49^ |
| Valvular regurgitation/insufficiency | 120 | da Costa et al.^33,40,46^; Etnel et al.^41^; Sievers et al.^49^ |
| Valvular stenosis | 106 | da Costa et al.^33,40,46^; Etnel et al.^41^; Ruzmetov et al.^53^; Sievers et al.^49^ |
| Infective endocarditis | 1 | Sarikouch et al.^34^ |
| Other ^‡^ | 230 | Bibevski et al.^48^; Boethig et al.^50^; Brown et al.^45^; Cebotari et al.^43^; da Costa et al.^33^; Ruzmetov et al.^53^; Sarikouch et al.^34^; Sievers et al.^49^ |
| ^†^ Congenital heart disease encompassed the following conditions: Tetralogy of Fallot (n = 209); truncus arteriosus communis (n = 40); pulmonary atresia with an intact ventricular septum (n = 38); transposition of the great arteries (n = 26); pulmonary atresia with a ventricular septal defect (n=24); double outlet right ventricle (n = 16); transverse aortic constriction (n =2); and was not specified in 187 cases.  ^‡^ Other conditions included: acquired heart diseases (n = 146); Ross procedure (n = 55); congenital and acquired heart diseases (n = 2); dysfunction of heart valve prosthesis (n = 1); and was not specified in 26 cases. | | |
