# Supplementary material for: Clinical performance of decellularized heart valves versus standard tissue conduits: a systematic review and meta-analysis
Source: J Cardiothorac Surg. 2020 Sep 18;15:260. doi: 10.1186/s13019-020-01292-y (PMC7501674; doi:10.1186/s13019-020-01292-y)
Supplement: Supplementary file 5 — Additional file 5. Primary endpoints (postoperative mortality and reoperations) in eligible full-text articles. [file 13019_2020_1292_MOESM5_ESM.docx]

| **Additional File 5.** Primary endpoints (postoperative mortality and reoperations) in eligible full-text articles | | | | | | |
| --- | --- | --- | --- | --- | --- | --- |
| **Authors** | **Primary endpoints**^†^ | | | | | |
|  | **Mortality (%)** | | | **Reoperations (%)** | | |
|  | **30-day** | **1-year** | **Overall** ^‡^ | **1-year** | **Overall** ^‡^ | **Time to reoperation (years)**^§^ |
| Bechtel et al. 2005 ^39^ |  |  |  |  |  |  |
| Bechtel et al. 2008 ^42^ |  |  |  |  | 0 (0.0) vs. 1 (2.0); P = 0.46 |  |
| Bibevski et al. 2017 ^48^ | 8 (4.9) vs. 5 (4.0) |  | 15 (9.2) vs. 13 (10.5); P = 0.72  Freedom from mortality: 94% vs. 94%; P = 0.84 |  | 16 (9.8) vs. 32 (25.8)  Freedom from reoperations higher in decellularized group (P = 0.001) | 3.6 ± 2.2 vs. 3.1 ± 2.7;  P = 0.51 |
| Boethig et al. 2019 ^50^ | 2 (0.9) vs. 0 (0.0) |  | 2 (0.9) vs. 0 (0.0);  Freedom from mortality: 98.9% vs. 97.4%; P = 0.12 |  | 2 (0.9) vs. 0 (0.0) |  |
| Brown et al. 2010 ^45^ | 7 (2.0) vs. 75 (6.0) | 1 (0.3) | 17 (5.0) vs. 113 (9.1)  Freedom from mortality: 92.3% vs. 84.6%, P = 0.009 and 96.9% vs. 94.8%; P > 0.05 (Isolated RVOT reconstruction and Ross procedure respectively) |  | 22 (6.4) vs. 91 (7.3) Freedom from reoperations: 92.7% vs. 89.5%;  P > 0.05 and 94.4% vs. 95.5%, P > 0.05  (Isolated RVOT reconstruction and Ross procedure respectively) |  |
| Brown et al. 2011 ^47^ | 0 (0.0) vs 0 (0.0) |  | 0 (0.0) vs. 0 (0.0) |  | 1 (3.4) vs. 2 (5.9); P = 0.68 |  |
| Burch et al. 2010 ^51^ |  |  | 2 (4.3) vs. 1 (2.1) |  | 9 (19.1) vs. 13 (27.7)  Freedom from reoperations: 79% vs 63%;  P = 0.31 |  |
| Cebotari et al. 2011 ^43^ | 0 (0.0) | 1 (2.6) | 1 (2.6) vs. 0 (0.0) |  | 0 (0.0) vs. 11 (14.5)  Freedom from reoperations: 100% vs. 87.6% |  |
| da Costa et al. 2005 ^46^ | 0 (0.0) |  | 0 (0.0) vs. 0 (0.0) |  | 1 (9.1) vs. 0 (0.0) |  |
| da Costa et al. 2007 ^33^ | 1 (1.5) |  | 2 (2.9) vs. 0 (0.0) | 1 (1.5) | 2 (2.9) vs. 0 (0.0) |  |
| da Costa et al. 2018 ^40^ | 1 (1.1) vs. 0 (0.0); P = 1.00 |  | 4 (4.3) vs. 4 (4.3)  Freedom from mortality: 95.3% vs. 97.7%; P = 0.323 |  | 2 (2.1) vs. 10 (10.6)  Freedom from reoperations: 98.8% vs. 95.5%;  P = 0.383 |  |
| Etnel et al. 2018 ^41^ | 3 (2.3) vs. 2 (1.5) |  | 3 (2.3) vs. 14 (10.8); P = 0.759 |  | 1 (0.8) vs. 8 (6.2); P = 0.642;  Freedom from reoperations: 99.2% vs. 97.6%; P = 0.642 |  |
| Konuma et al. 2009 ^52^ | 4 (9.8) vs. 3 (7.3) |  | 6 (14.6) vs. 6 (14.6) |  | 3 (7.3) vs. 8 (19.5); P = 0.10  Freedom from reoperations: 86% vs. 76%; P = 0.29 |  |
| Ruzmetov et al. 2012 ^53^ | 1 (2.6) vs. 2 (3.3) |  | 5 (12.8) vs. 6 (9.8), P = 0.51;  Freedom from mortality: 92% vs. 89%;  P = 0.75 |  | 5 (12.8) vs. 19 (31.1) | 3.4 ± 3.1 vs. 3.4 ± 2.8; P = 0.96 |
| Sarikouch et al. 2016 ^34^ |  |  | 2 (2.2) vs. 0 (0.0);  Freedom from mortality: 97.8% vs. 95.5% P > 0.05 |  | 1 (1.1) vs. 0 (0.0) |  |
| Sievers et al. 2003 ^49^ |  | 1 (5.9) | 1 (5.9) vs. 0 (0.0) |  |  |  |
| Tavakkol et al. 2005 ^54^ | 3 (11.5) vs. 2 (7.7) |  | 4 (15.4) vs. 4 (15.4) |  | 1 (3.8) vs. 2 (7.7); P = 0.98 |  |
| Blank cells correspond with data points that were not reported in their respective article.  n, number; P, probability value; RVOT, right ventricular outflow tract; SD, standard deviation; vs, versus.  ^†^ All results are recorded in the format: Decellularized heart valve cohort vs standard tissue conduit cohort. In cases where isolated results are shown, this relates to the decellularized heart valve cohort only.  ^‡^ Freedom from mortality and freedom from reoperations reported at a follow-up period of five years in all cases.  ^§^ Values reported as mean ± SD. | | | | | | |
