# Supplementary figures and images for: Clinical performance of decellularized heart valves versus standard tissue conduits: a systematic review and meta-analysis
Source: J Cardiothorac Surg. 2020 Sep 18;15:260. doi: 10.1186/s13019-020-01292-y (PMC7501674; doi:10.1186/s13019-020-01292-y)

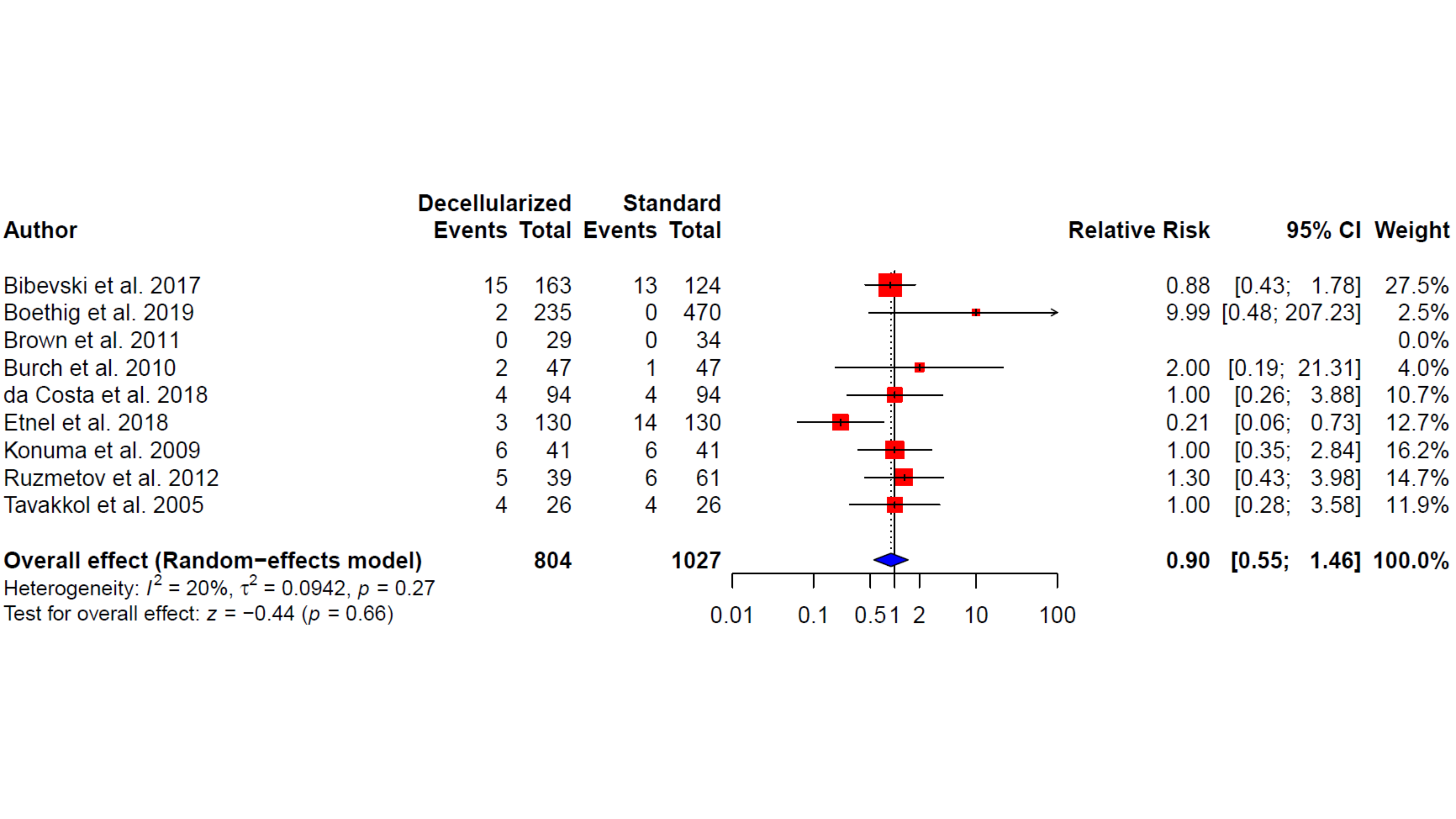

Supplement: Supplementary file 6 — Additional file 6. Forest plot of postoperative mortality rates following outflow tract reconstruction surgery with decellularized heart valves versus standard tissue conduits. Studies at moderate or serious risk of bias and with a disproportionately large sample size have been excluded for the purposes of this sensitivity analysis. Pooled summary estimates are shown as relative risks (RR) with their 95% confidence intervals (CI). [file 13019_2020_1292_MOESM6_ESM.tif]

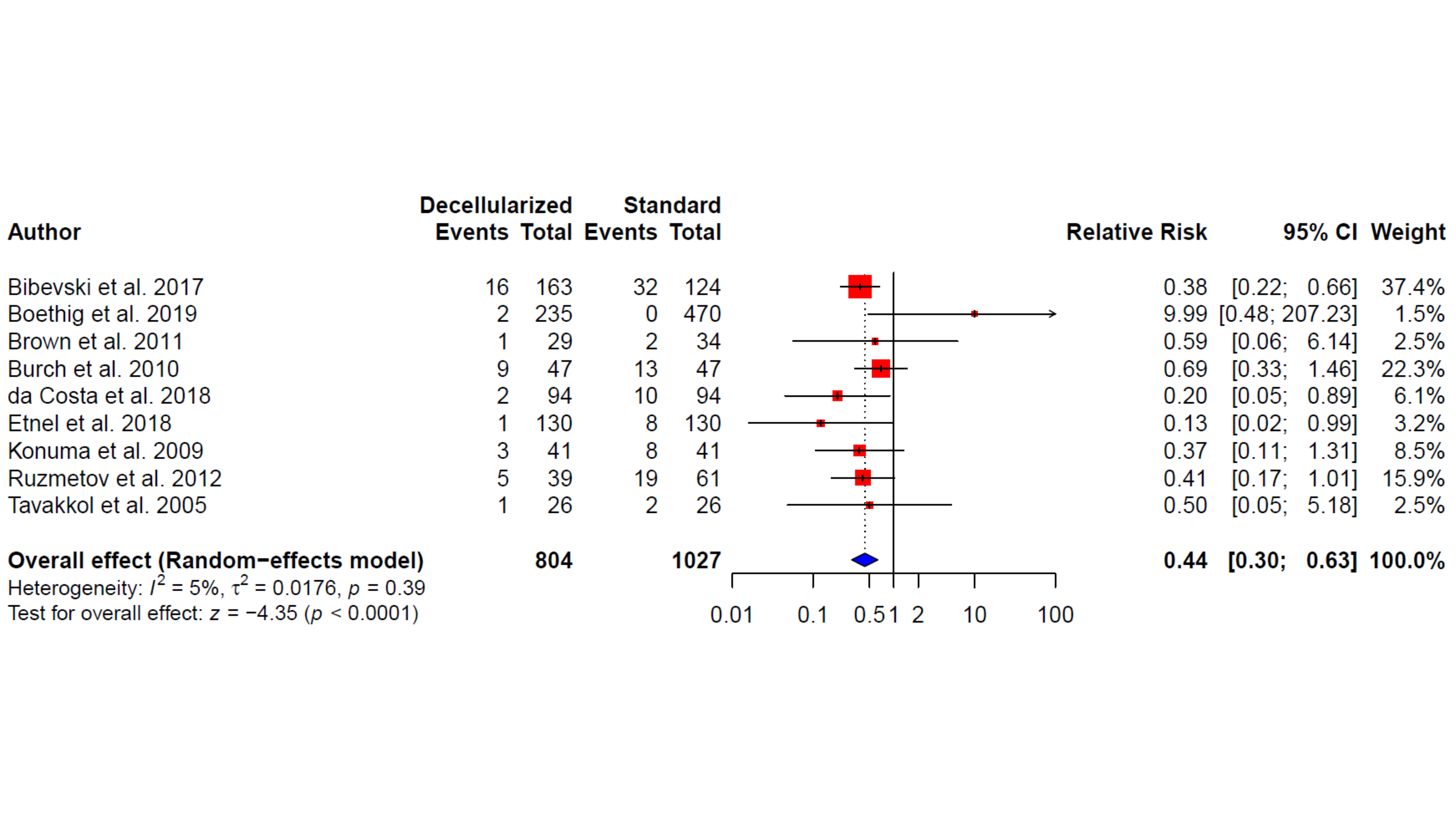

Supplement: Supplementary file 7 — Additional file 7. Forest plot of reoperation rates following outflow tract reconstruction surgery with decellularized heart valves versus standard tissue conduits. Studies at moderate or serious risk of bias and with a disproportionately large sample size have been excluded for the purposes of this sensitivity analysis. Pooled summary estimates are shown as relative risks (RR) with their 95% confidence intervals (CI). [file 13019_2020_1292_MOESM7_ESM.tif]
